# Healing Response to Hyaluronic Acid Supplemented with Selected Amino Acids: A Pooled Analysis of Two Clinical Studies

**DOI:** 10.3390/jfb17080354

**Published:** 2026-07-24

**Authors:** Tiziana Ruggiero, Marta Bezzi, Ettore Cogno, Davide Camisassa, Renato Pol, Francesco Erovigni, Giorgio Reggiardo, Vincenzo Nobile, Paolo Giacomo Arduino

**Affiliations:** 1 Department of Surgical Sciences, C.I.R. Dental School, University of Turin, Via Nizza 230, 10126 Turin, TO, Italy; 2 Department of Mechanical and Aerospace Engineering, Politecnico di Torino, Corso Duca degli Abruzzi 24, 10129 Turin, TO, Italy; 3 Department of Biostatistics, Clinical Validation from Biopharmaceutical Findings (CVBF), Viale Cesare Battisti 17, 27100 Pavia, PV, Italy; 4 Research & Development Department, Complife Italia S.r.l., Via Indipendenza 11, 27100 Pavia, PV, Italy

**Keywords:** amino acids, diabetes mellitus, type 2, hyaluronic acid, individual participant data, pooled analysis, randomized controlled trial, tooth extraction, wound healing

## Abstract

Background/Objectives: Diabetes is associated with impaired wound healing, with consequences for tissue recovery across surgical procedures. In the oral context, hyaluronic acid-based adjunctive therapies have shown promise in improving early post-extraction healing in diabetes; however, clinical evidence remains limited. Different supplemented HA-based formulations yield heterogenous healing outcomes. Moreover, whether age or diabetes duration influences the effect of these adjunctive therapies remains to be established. Methods: We conducted an individual-participant-data pooled analysis of two randomized controlled trials that evaluated the use of hyaluronic acid supplemented with two different sets of selected amino acids; individual data from each trial was combined into a single dataset. Results: In this pooled analysis, modified healing index scores improved significantly over time. When each intervention was evaluated relative to its corresponding control, hyaluronic acid plus six pro-collagen synthesis amino acids produced a significantly greater improvement in healing response over time than the other HA-based formulation, after adjustment for age (Greenhouse–Geisser: time × treatment: *F* = 5.821, *p <* 0.001) and diabetes duration (Greenhouse–Geisser: time × treatment: *F* = 4.939, *p* < 0.001). Neither age nor diabetes duration significantly modified the healing trajectory (Greenhouse–Geisser: time × age: *F* = 2.536, *p* = 0.083; time × diabetes duration: *F* = 0.131, *p* = 0.878). No clear association with age or diabetes duration was observed. Conclusions: Supplementing hyaluronic acid with six selected amino acids associated with a significantly faster post-extraction tissue recovery in patients with diabetes. Moreover, treatment choice was relevant regardless of age or years since diabetes diagnosis.

## 1. Introduction

Major lifestyle transitions in recent decades have broadened the risk for diabetes worldwide, particularly type 2 diabetes mellitus (T2DM), making this condition a major global public health concern [1,2,3]. The increase in diabetes prevalence occurs as part of a wider cluster of interrelated cardiometabolic conditions. These include obesity, metabolic liver disease, kidney disease, and hypertension, all of which together contribute to the growing burden of cardiovascular disease and exert lifetime adverse effects on vascular health and tissue repair [4,5,6,7,8,9].

Macrovascular complications remain a leading cause of premature mortality in individuals with diabetes [10,11,12], and among this population, cardiovascular disease risk is markedly elevated [10]. The presence of chronic kidney disease or reduced kidney function in the presence of diabetes further amplifies cardiovascular mortality, which reduces the life expectancy of this population [13,14]. Furthermore, microvascular and systemic complications in diabetes contribute substantially to disability and loss of quality of life, with far-reaching consequences for vascular health, immune function, inflammation, and tissue repair, directly impairing post-surgical wound healing [15,16,17]. Consequently, diabetes is increasingly being recognized as a systemic factor that negatively influences perioperative outcomes and postoperative recovery across all surgical disciplines, by hindering tissue repair and increasing the risk of infection [18,19,20,21].

Research on diabetic wound pathophysiology describes a hyperglycemic milieu characterized by endothelial dysfunction, impaired angiogenesis, dysregulated inflammation, oxidative stress, and increased susceptibility to microbial colonization [22,23,24,25]. These abnormal processes provide a mechanistic basis for delayed postoperative healing in patients with diabetes [23,26]. Therefore, perioperative assessments increasingly incorporate measuring of glycemic indices and assessing diabetes-related complications, both of which relevant to postoperative recovery [18,20].

Among surgical specialties, these concerns also apply to oral surgery [27,28]. In the oral cavity, the pathophysiological disruptions that are often associated with diabetes manifest through analogous local mechanisms that mirror the ones observed in other wound healing contexts, therefore predisposing patients with diabetes to postoperative complications during oral and maxillofacial surgery [27,29]. Experimental evidence indicates that diabetic wound healing of the oral cavity is marked by persistent inflammation, impaired epithelial and connective-tissue regeneration, oxidative stress, disrupted bone-remodeling, and an altered microbiome [27,30,31], all of which translate into increased risk of periodontal disease and tooth loss [27,29,32,33]. Together these alterations offer a mechanistic basis for the delayed recovery of both soft and hard tissues observed following dento-alveolar procedures [28,34]. Some studies have examined socket healing as short-term postoperative outcome following oral surgical interventions in patients with T2DM [29,35,36]. Clinical results suggest that diabetes may be associated with delayed socket healing in the early stages of healing, and increased postoperative symptom burden [28,29,37]. These findings are broadly consistent with pathophysiological processes of the oral cavity in diabetes, although the degree of socket healing impairment appears to depend on glycemic control and diabetes disease burden [28,37,38].

Therapeutic research on strategies intended to mitigate the adverse effects of diabetes on oral tissue repair is limited and shows mixed results [27,35,36,39]. Much of the work is based on broader diabetic wound healing studies, not only of the oral cavity, in which biomaterials have been investigated for their capacity to support the wound microenvironment while facilitating the local delivery of formulations supplemented with bioactive agents that may enhance granulation, re-epithelialization, and tissue remodeling [40,41,42]. Systemic adjunctive approaches, such as nutritional supplementation, have also been explored in some of these broader diabetic wound settings [43,44,45].

In particular, hyaluronic acid (HA) has attracted interest because of its relevance in wound healing across different surgical contexts in the presence of diabetes [40] and in post-tooth extraction in healthy individuals [46,47,48]. Beyond its established physico-chemical role in maintaining tissue hydration, preclinical and clinical evidence support a role for hyaluronic acid in improving early post-extraction tissue healing [46,47,48,49]. However, evidence from oral-surgical settings in patients with diabetes is scarce; local hyaluronic acid-based topical formulations appear promising for improving early mucosal healing after tooth extraction in this population, providing a rationale for further evaluation of targeted perioperative approaches [35,36,50]. Randomized studies in post-extraction populations have reported improved early postoperative outcomes with hyaluronic acid-based interventions [35,50]. However, whether treatment effects differ between formulations and to what extent they are influenced by patient characteristics such as age and diabetes burden remains confounded by the mixed clinical findings [35,36,50,51].

In diabetes, hyaluronic acid has also been evaluated in combination with adjunctive wound healing agents designed to optimize the local healing environment while promoting early postoperative recovery following oral surgery [36,52]. Findings from these combination approaches cannot be attributed to hyaluronic acid alone but rather to their combined action with such bioactive supplements [36,52]. Further studies are required to clarify the effects of these novel adjunctive formulations across different population profiles. Accordingly, indicators of diabetes disease burden should be considered in both study design and statistical analysis [28,35,36,39].

Two previous clinical studies in patients with T2DM reported that topical HA-based therapies exhibited significantly improved early healing outcomes after tooth extraction relative to untreated controls [35,36]. Differences between the two formulations and variations in follow-up healing-index values provided the rationale for the present study, which was designed to evaluate HA alone and HA in combination with two distinct pools of selected amino acids, while adjusting for age and duration of diabetes [35,36]. The amino acids included provide local substrates relevant to extracellular-matrix formation and collagen synthesis during wound repair [35,36].

In the present study, we conducted a pooled analysis of individual participant data from two randomized controlled trials [35,36] to compare the longitudinal post-extraction healing trajectories associated with two new hyaluronic acid-based formulations containing different amino acid combinations, while also assessing whether age and diabetes duration modified the treatment response of these two formulations in patients with T2DM.

## 2. Materials and Methods

### 2.1. Ethics Approval and Informed Consent

The two studies were conducted in accordance with the ethical principles of the Declaration of Helsinki and its subsequent revisions (Ethical Principles for Medical Research Involving Human Subjects, adopted by the 18^th^ WMA General Assembly Helsinki, Finland, June 1964, and amendments). The study protocols and all related research procedures were reviewed and approved by the local ethics committee of the University of Turin (with code 0100924 on 15 September 2022). Before enrollment, all participants were fully informed about the aims of the research and provided written informed consent before the initiation of any study procedures. The reporting of the trials was prepared in accordance with the CONSORT 2010 and CONSORT 2025 statements. Retrospective ClinicalTrials.gov registration ID: NCT05896319, registration date: 9 June 2023.

### 2.2. Study Design and Participants

The present study was designed as an individual participant data pooled analysis of two previous randomized controlled trials conducted at the C.I.R. (Interdepartmental Research Center), Dental School, Section of Oral Surgery, Department of Surgical Sciences, University of Turin (Italy). Both studies were single-center trials involving patients with type 2 diabetes mellitus requiring extraction of at least one non-impacted tooth [35,36]. The first trial, conducted between September 2022 and February 2023, was designed as a split-mouth randomized study. It included patients requiring bilateral extraction of homologous, non-impacted teeth [35]. The second trial, conducted between September 2023 and February 2024, followed a two-arm, parallel-group randomized controlled design. Participants were allocated either to the topical gel intervention group or to a control group receiving no adjunct treatment [36].

Inclusion criteria for both studies were age of 18 years or older with a previous diagnosis of type 2 diabetes mellitus, documented history of diabetes-related complications, requiring extraction of non-impacted teeth, having signed informed consent, and with availability for follow-up visits. Diabetes-related complications included nephropathy, neuropathy, retinopathy, cardiopathy/cardiomyopathy, or peripheral vascular disease. Exclusion criteria for both studies included platelet dysfunction, thrombocytopenia, corticosteroid therapy, current smoking, refusal to participate, poorly controlled diabetes mellitus, and use of medications known to interfere with wound healing. Additional exclusion criteria common to both studies were extractions requiring flap elevation, teeth requiring sectioning with burs, ankylosed teeth requiring bur-assisted extraction, and apical root fractures occurring during the extraction procedure. Randomization in both trials was performed using a computer-generated random sequence created with SPSS version 24.0 (SPSS Inc., Chicago, IL, USA). Before enrollment, all participants were fully informed about the study procedures and provided written informed consent prior to any study-related procedures.

### 2.3. Interventions and Procedures

Participants in both trials received topical adjunctive treatment following tooth extraction. In the first trial, the intervention consisted of Aminogam^®^ gel (Professional Dietetics S.p.A., Milan, Italy), a formulation containing hyaluronic acid and four synthetic amino acids, including glycine, L-proline, L-leucine, and L-lysine hydrochloride, together with purified water and other excipients. In the second trial, the intervention consisted of Aminogam^®^ 6 gel (Professional Dietetics S.p.A., Milan, Italy), a topical hyaluronic acid-based formulation and six synthetic amino acids, including glycine, L-proline, L-leucine, L-lysine HCl, L-valine, and L-alanine. Both interventions were administered after tooth extraction according to the study protocol [35,36].

Before tooth extraction, all participants underwent a professional oral hygiene session, followed by clinical and radiographic evaluation to collect baseline data [35,36]. Demographic variables included sex, age, ethnic origin, body mass index, and smoking status, while diabetes-related variables comprised duration of diabetes, glycemic status on the day of surgery, glycated hemoglobin (HbA1c), and end-organ disease score [35,36]. The preoperative dental assessment included the number of roots, the presence of caries, pulp vitality, previous endodontic treatment, and the presence of periapical lesions. In both studies, extraction difficulty was classified as low, intermediate/medium, or high based on clinical and radiographic parameters, and systemic risk was evaluated using a structured classification based on diabetes-related clinical features [35,36].

All surgical procedures were performed according to the described protocols; both extractions were performed under local anesthesia. After surgery, all participants received standardized postoperative instructions and oral hygiene recommendations. Patients allocated to the test group were instructed to apply the assigned topical gel locally three times daily for 7 days according to the respective study protocol [35,36]. Anti-inflammatory drugs and antibiotics were not routinely prescribed in either study, and postoperative antibiotic use was considered an indicator of complications related to infection.

### 2.4. Clinical Outcomes

Post-extraction socket healing was assessed using a modified Landry healing index as the primary clinical outcome [17,36]. Because the original Landry index was developed for wound healing by primary intention, it was adapted for the assessment of extraction sockets, which heal by secondary intention through granulation tissue formation and subsequent epithelialization [17,36]. In the modified scoring system, healthy granulation tissue was regarded as a favorable feature of early tissue repair rather than as an adverse clinical finding [17,36]. The modified index comprised four clinical domains: tissue color, bleeding, granulation tissue, and suppuration [17,36]. Each domain was scored from 1 to 3, and the individual scores were summed to obtain a total score ranging from 4 to 12. Lower total scores indicated a more favorable clinical appearance and more advanced healing, whereas higher scores indicated poorer healing. Tissue color was scored as 1 when the gingiva appeared entirely pink, 2 when red or hyperemic tissue involved no more than 50% of the assessed area, and 3 when these changes involved at least 50% of the area. Bleeding was scored as 1 when absent, 2 when elicited by palpation, and 3 when spontaneous. Granulation tissue was scored as 1 when pink, firm, and finely granular, 2 when red and soft, and 3 when friable, greenish, or grayish. Suppuration was scored as 1 when no plaque was present at the socket margins, 2 when plaque accumulation was visible along the alveolar walls, and 3 when suppuration or clinical signs of alveolitis were observed [17,36].

### 2.5. Statistical Analysis

Continuous data were expressed as mean, standard deviation, median, minimum and maximum. Categorical data were described using frequencies and percentages. Comparisons between treatment groups of categorical data were made using the chi-square test or Fisher’s exact test. The Z test was used to test for differences in binomial proportions. Comparisons between treatment groups of continuous data were made using the Analysis of Variance (ANOVA). To compare the trends in outcome indicators over time in the groups of patients at Day 3, Day 7, Day 14 and Day 21, normally distributed variables were examined using repeated-measures ANOVA. The test for significance was a between-groups comparison (difference between treatment groups within study visit). Comparing treatment groups at individual visits is appropriate for assessing if the groups differ at that specific time-point. A General Linear Model (GLM) repeated-measures ANOVA was done with the within-subjects factor being time/assessments at four levels (Day 3, 7, 14, and 21) and the between-subjects factor being treatment groups at two levels (AM4 vs. AM6). Both group-by-time interaction effects and between-subjects and within-subjects effects were assessed. Planned contrasts for the within-subject factor “time” were used to test specific a priori hypotheses about how the dependent variable changes over time (Day 3 vs. Day 7, Day 14, and Day 21).

Significance tests were two-sided. A *p*-value of <0.05 for all tests was considered statistically significant. Data were entered and analyzed using SPSS version 29.0 (IBM Corporation, Armonk, NY, USA).

## 3. Results

### 3.1. Baseline Characteristics

The present study analyzed pooled individual participant data from two trials to evaluate post-extraction healing trajectories between two formulations containing hyaluronic acid and selected amino acids. The models used accounted for age and years since diabetes diagnosis, and incorporated both within- and between-subject comparisons. At baseline, the pooled cohort included 112 participants, evenly distributed by sex (57 males, 55 females). All participants were Caucasian; the mean age was 69.44 ± 10.08 years. Additional baseline characteristics included diabetes duration, smoking status, BMI, preoperative glycemic status, HbA1c, diabetes-related end-organ complications, and systemic risk classification (Table 1). Across both trials, all enrolled participants completed follow-up and were included in the efficacy and safety analyses. Both topical interventions were well tolerated, with no reported adverse reactions, treatment discontinuations, or requirement for antibiotic therapy during the study periods.

Group baseline descriptive statistics, including age, sex distribution, BMI, and years since diabetes diagnosis by study group, are provided in the Supplementary Materials. Between-group comparisons were assessed using ANOVA for continuous variables and chi-square tests for categorical variables (Tables S1–S4).

### 3.2. Healing Index over Time and Across Cohorts

Table 2 presents the descriptive statistics for the modified healing index (mHI) at each follow-up visit (Day 3, Day 7, Day 14 and Day 21), for the two intervention groups and their matching control groups. Lower scores denote more favorable healing; thus, the progressive reduction in mean and median values over time indicates continued improvement in extraction-socket soft-tissue repair. For each group and time point, the table shows the number of participants (N) as well as the mean, standard deviation, median, and minimum and maximum mHI values. Data displays healing progression over time and across cohorts. Sockets treated with Aminogum^®^/Aminogam^®^ 6 Gel (AM6) displayed a statistically significant difference in modified healing index values at days 7 (*p* = 0.01) and 14 (*p* = 0.02) (* *p*-value < 0.05, AM6 vs. C6) and had already reached optimal healing by day 7 (Table 2). In contrast, sockets in both control groups only achieved comparable healing by day 21 (Table 2).

To investigate differences in the healing response across the two treatments post-extraction, we conducted a univariate one-way ANOVA comparing AM6 with AM4 on the modified healing index scores recorded over the four follow up visits. We failed to observe any statistically significant difference in healing between the two interventions on day 3. By day 7, however, patients receiving AM6 showed significantly better healing scores than those in AM4, suggesting faster healing response in the AM6 compared to the AM4 cohort (*p* < 0.01).

Healing outcomes were similar between groups on the third follow-up visit. This finding indicates that both cohorts eventually reached comparable healing outcomes (Table 3). Overall, these data suggest that treatment with AM6 intervention accelerates the healing process compared to AM4, with patients in this group showing evidence of socket healing as early as day 7; complete healing was observed in both treatment groups by day 14 (Table 3).

Besides treatment allocation, progress in healing index scores can be confounded by patient demographic and clinical characteristics such as age and diabetes duration. Importantly, we observed a statistically significant difference in mean age and years since diabetes diagnosis between cohorts at baseline (Tables S1 and S4 in the Supplementary Materials). To investigate whether these two characteristics could influence healing outcomes, we carried out repeated-measures general linear models (GLM) adjusted for age or years since diagnosis, and modeled within-subject and between-subject variations in healing index scores over time and across the two treatment groups, first using treatment scores (AM6 vs. AM4) alone, and then fitting healing scores expressed relative to each corresponding control group (AM6 relative to C6 vs. AM4 relative to C4).

### 3.3. Age-Adjusted Analysis of AM6 vs. AM4 Response on Socket Healing: Within- and Between-Subject Comparisons

We initially assessed the within-subject effect of time by pooling together the two treatment groups, AM6 and AM4, over the course of the four follow-up visits. We evaluated whether the sphericity assumption was maintained by using the Mauchly’s test. For time, Mauchly’s W was 0.098, with an approximate chi-square value of 120.062 and a *p*-value < 0.001, indicating a violation of sphericity. Consequently, F-tests for time-related effects were based on corrected degrees of freedom derived from the Greenhouse–Geisser, Huynh–Feldt, and lower-bound epsilon estimates, as reported in Table 4A.

We next examined changes in healing index scores over the four follow-up visits with time as the within-subjects factor, treatment (AM6 vs. AM4) as the between-subjects, and age entered as a covariate. Because Mauchley’s test indicated that the assumption of sphericity was violated for time, corrected statistics are reported. Using Greenhouse–Geisser, Huynh–Feldt, and lower-bound-corrected tests, the analysis shows that the effect of time was not significant after adjusting for age, when the two treatments were pooled over the four follow-up visits. The time × age interaction showed a trend without reaching significance (*p* = 0.052, Greenhouse–Geisser correction), indicating that any age-related effect on the healing index progression across time was weak and should be interpreted with caution (Table 4B). Notably, the time × treatment interaction was statistically significant after corrections (*p* < 0.05), demonstrating that the progression of post-extraction socket healing over time significantly differed between the two treatments after adjusting for age. Clinically, these results suggest that, although participants across cohorts achieved a comparable level of healing by the final visit (Table 2 and Table 3), their socket healing patterns diverged significantly over time, with the AM6 cohort recovering at earlier time points compared with the AM4 cohort (Table 4B). These findings indicate that the choice of treatment influences the speed of post-extraction socket healing response after accounting for the age of the participants (Table 2 and Table 4B).

To further characterize change in healing index over time, we conducted within-subjects contrast analyses with time (across the four visits) as the repeated factor, AM6 and AM4 treatments as the between-subjects factor, and age as a covariate (Table 4C). We could not observe a significant overall linear effect of time when both treatments were pooled. Likewise, the time × age linear contrast displayed no significance, meaning that age did not systematically influence the linear rate of response in healing index over time. By contrast, the time × treatment linear trajectory was significantly different across the two interventions (*p <* 0.05). This finding is consistent with AM6 showing a faster recovery in the post-extraction socket healing than AM4 and provides further evidence that the choice of treatment influences how quickly patients recover over the follow-up period, even though both groups ultimately obtain a similar level of healing (Table 2 and Table 4).

In the between-subjects test, treatment again displayed a significant effect on the overall healing response after adjusting for age (*p <* 0.01), indicating that the two interventions differed in average healing response across the follow-up period. Age showed a non-significant trend (*p =* 0.064), suggesting that although age did not meaningfully influence the overall outcome once treatment assignment was accounted for, a tendency was nonetheless observed (Table 4D).

We next examined whether variations between the two interventions in the course of healing persisted when each treatment was considered relative to its corresponding control, taking age as a covariate. To this end, we generated a GLM repeated-measures model comparing the healing response between the two treatments relative to their controls over time. Similarly to the previous analyses, we took time as the within-subjects factor and age as a covariate. Because Mauchly’s test indicated a violation of the sphericity assumption (W = 0.364), corrected statistics were used for all within-subjects tests in the subsequent analyses of the treatment relative to control, as reported in Table 5A (Table 5).

As shown in Table 5B, the effect of time when pooling both treatments together (relative to their matching controls) was not statistically significant after correction for sphericity. Consistent with the previous analysis, the time × age interaction also failed to reach significance under any of the corrections (*p =* 0.083, Greenhouse–Geisser). By contrast, the time × treatment interaction (relative to controls) displayed significant variations in healing progression under all corrections (*p* < 0.001, Greenhouse–Geisser), indicating that the progression of healing over time differed significantly after adjusting for age (Table 5B). For the within-subjects contrast test, the time × age linear interaction was significant (*p <* 0.05), suggesting that age showed a within-subjects association with systematic variation during the course of healing. Most importantly, the time × treatment linear effect was statistically significant at the *p* < 0.001 level, further indicating that the treatment effects of the two interventions relative to their controls diverged over time in a linear manner. This observation is consistent with the different rates of healing in response to each intervention (Table 5C).

For the between-subjects analysis, both age and treatment relative to corresponding control displayed significance as predictors of healing response. The effect of treatment relative to control was statistically significant at the *p <* 0.001 level, further confirming that the treatment response values relative to control strongly differed between the two interventions even after adjusting for age. In addition, the participant’s age showed a statistically significant effect on healing response, albeit at a lower level of significance (*p <* 0.05), suggesting age exerts a more modest influence on healing compared with treatment relative to control (Table 5D).

### 3.4. AM6 vs. AM4 Healing Outcomes Adjusted for Years Since Diabetes Diagnosis: Within- and Between-Subject Comparisons

We next conducted repeated-measures general linear modeling (GLM), taking the responses of the two intervention groups only (AM4 vs. AM6), with time as the within-subjects factor, the two interventions as the between-subjects factor, and years since diabetes diagnosis (T2DM Dx) as a covariate. The Mauchly’s test indicated violation of sphericity for time (W = 0.112); Greenhouse–Geisser, Huynh–Feldt, and lower-bound corrections are reported (Table 6A).

Similarly to previous analyses, we re-ran the repeated-measures GLM with years since diabetes diagnosis instead of age as the covariate and the within-subjects contrasts. In this model, the time × treatment interaction no longer reached statistical significance (*p =* 0.078, Greenhouse–Geisser correction), although we could observe a differential tendency in the healing trajectory across both interventions (Table 6B). These results show that the healing differential response between interventions in the time x treatment interaction is modest without reaching significance at a statistical level when adjusting for years since diabetes diagnosis (T2DM Dx) (Table 6B).

However, the between-subjects analysis showed a significant variation in the course of healing depending on the choice of treatment (*p <* 0.05), whereas years since diabetes diagnosis was not associated with different healing trajectories. Thus, when averaged across time points, healing differed between the two intervention groups independently of number of years since diabetes diagnosis (Table 6D), suggesting a similar pattern of improvement for both intervention groups over time, albeit with different average healing scores (Table 6C,D). This analysis only took the response to treatment in healing. We next compared the trajectories in healing response between the two interventions relative to their respective controls.

To account for the controls, we repeated the GLM analysis across both intervention groups relative to their corresponding controls (AM6 relative to C6 vs. AM4 relative to C4), with years since diagnosis (T2DM Dx) as covariate. For this model, the Mauchly’s test for the within-subjects factor time again indicated departure from sphericity (W = 0.375); consequently, corrected factors and *p*-values are reported for time (Table 7A).

When treatment healing outcomes were expressed relative to their corresponding control groups, the repeated-measures GLM again showed a statistically significant variation effect of time on healing progression, further supporting that the treatment healing rate systematically changed relative to the control across the four follow-up visits. The time × T2DM Dx interaction remained non-significant (*p >* 0.05), suggesting that diabetes duration did not influence healing response over time. In contrast, the time x treatment interaction of both treatments relative to controls was statistically significant at the *p <* 0.001 (Greenhouse–Geisser-corrected), suggesting that the healing responses were significantly different over time when analyzed relative to their respective controls and after adjusting for years since diabetes diagnosis (Table 7B).

Analysis of within-subjects contrasts confirmed a linear effect of time (*p <* 0.001), indicating a change in the healing trajectory relative to controls across time points assessments. The time × T2DM Dx (expressed in years since diabetes diagnosis) interaction remained unrelated to healing response (*p >* 0.05), whereas the linear time x treatment interaction became significant at the level of *p <* 0.001, further supporting a significantly differential linear healing progression when comparing the two treatments relative to controls over time (Table 7C).

In the between-subjects test, when treatment outcomes were expressed relative to outcomes in control groups, years since diabetes diagnosis was again unrelated to healing response (*p >* 0.05), whereas treatment showed statistically significant different healing trajectories when comparing the two treatments, AM6 vs. AM4, at a level of significance of *p <* 0.001. Thus, when treatment healing index outcomes were expressed relative to control groups and adjusted for years since diabetes diagnosis, the observed healing trajectories were statistically different between the two interventions (Table 7D).

Overall, these findings mirror the results obtained in the models produced earlier using age as the covariate. Both age and years since diabetes diagnosis (T2DM Dx) have no significant effect on the healing trajectory over time. Variations in treatment healing responses are most evident when healing outcomes are reported relative to their respective controls. While the age-adjusted model exhibited a possible tendency for age to modulate the time x treatment interaction, years since diabetes diagnosis showed no such tendency, providing no evidence that either diabetes duration or early diagnosis may influence how the healing trajectory responds to the two interventions. Thus, treatment choice remained significantly relevant, with no clear evidence that its effect was modified by age or years since diabetes diagnosis.

## 4. Discussion

The present pooled analysis compared post-extraction socket soft-tissue healing trajectories between two hyaluronic acid-based formulations containing different amino acid combinations in patients with T2DM and analyzed whether age or diabetes duration influenced the healing response over time [35,36]. Across the follow-up period, modified healing index scores improved for both intervention groups. Further, when each intervention was evaluated relative to its corresponding control, AM6 showed a significantly greater improvement in healing over time than AM4 after adjustment for age (Greenhouse–Geisser: time × treatment interaction: *F* = 5.821, *p* < 0.001) and diabetes duration (Greenhouse–Geisser: time × treatment: *F* = 4.939, *p* < 0.001). Neither age nor diabetes duration significantly modified the healing trajectory (time × age interaction: *F* = 2.536, *p* > 0.05; time × diabetes duration interaction: *F* = 0.131, *p* > 0.05). Clinically, the decrease in modified healing index scores reflects improvement in the soft-tissue characteristics of extraction-socket repair assessed by the healing index scores. The significant control-adjusted time × treatment interactions indicate that AM6 was associated with a more favorable healing trajectory than AM4, particularly during the earlier stages of follow-up. Thus, the time x treatment effect appears to have be an acceleration of clinical healing rather than a sustained difference in healing status at the end of the observation period, as the two intervention groups showed more comparable scores by Day 21. The biological mechanisms underlying this difference were not evaluated and remain to be established.

Underlying mechanistic pathways may provide a biological rationale for the earlier improvement observed with AM6; however, the mechanisms behind the differential healing trajectories were not directly evaluated in either source trial [35,36]. Hyaluronic acid is a constituent of the extracellular matrix and has been reported to support fibroblast migration and proliferation, regulate wound repair, promote angiogenesis, and modulate local inflammatory responses [35,36,39,40]. Although the mechanistic effects of the specific amino-acid combination used in the active intervention AM6 were not directly assessed, the healing trajectory observed for the AM6 cohort is compatible with a biological rationale whereby amino-acid supplementation may provide substrates for collagen biosynthesis and extracellular-matrix deposition, processes central to wound repair and subsequent soft-tissue remodeling [53,54]. Experimental evidence from cultured human skin fibroblasts further suggests that a mixture comprising the same six amino acids may promote molecular pathways associated with collagen biosynthesis and extracellular-matrix formation, with mTOR signaling implicated in these responses [55]. These complementary mechanisms may be particularly relevant in T2DM, in which impaired fibroblast activity, extracellular-matrix dysregulation, attenuated angiogenesis, persistent inflammation, and abnormal macrophage polarization may contribute to impaired oral wound and extraction-socket healing [23,27,34,39]. Nevertheless, these findings provide only formulation-level mechanistic support and do not establish the respective contributions of each individual amino acid nor confirm that the same mechanisms operate in post-extraction oral wounds. Thus, any interaction between hyaluronic acid and the six selected amino acids remains hypothetical because it was not evaluated in the present analysis or in either source trial [35,36].

From a clinical perspective, the greater early reduction in modified healing index scores observed with AM6, particularly at Day 7, indicates faster improvement in the socket soft-tissue, as captured by the dimensions in the modified healing index, which include tissue color, bleeding, granulation tissue, and suppuration [35,36]. Furthermore, the convergence of the two intervention groups by Day 21 suggests an acceleration of early socket healing rather than a persistent difference in soft-tissue healing status at the end of follow-up. This is important because earlier improvement may be clinically relevant in patients with T2DM, in whom delayed extraction-socket healing and postoperative infection are recognized concerns [29,37,38,39]. Nevertheless, postoperative pain, patient comfort, and oral-health-related quality of life outcomes were not assessed in the two source trials [35,36]. Previous clinical studies and systematic reviews of locally applied hyaluronic acid after tooth extraction have reported potential benefits for early soft-tissue healing and postoperative symptoms, including pain and swelling, although findings remain heterogeneous across formulations and study designs [46,47,48,50,56,57]. Across the two source trials, no adverse reactions, treatment discontinuations, need for antibiotic therapy, or postoperative infections were reported [35,36]. Future trials comparing the two active interventions should determine whether the earlier improvement in healing-index scores translates into clinically meaningful benefits using prespecified clinical thresholds and comparative measures of pain, infection, antibiotic use, and oral-health-related quality of life.

This clinical context is particularly relevant because type-2 diabetes is associated with impaired wound healing, including in oral soft and hard tissues and post-extraction sockets [23,27,29,37,39]. Evidence across several surgical contexts suggests that diabetes burden, particularly elevated HbA1c, may adversely affect postoperative outcomes, including surgical site infection risk; however, the benefits of stricter perioperative glycemic control remain inconsistent across clinical settings and demographics [18,19,21,58]. This heterogeneity across different studies is important in oral surgery because delayed extraction-socket healing and postoperative infection remain clinically relevant concerns in patients with diabetes [29,37,38], further highlighting the need to take age and diabetes duration into account. The results obtained in the present pooled analysis show that the choice of HA-based intervention is significantly associated with healing trajectory after adjusting for age and years since diabetes diagnosis. These results are relevant in the context of tooth extraction in T2DM individuals because controlled diabetes or glycemic control has not consistently been associated with impaired healing in previous studies; however, these studies failed to account for disease duration and other relevant parameters to T2DM [51,59]. Furthermore, some studies have reported delayed extraction-socket healing in diabetic patients compared with non-diabetic controls [29,37]. These inconsistencies point to the need for further research that considers diabetes burden when evaluating postoperative recovery in diabetic populations [18,20]. In this context, the observed divergent healing trajectories depending on treatment choice are consistent with the existing literature. In non-diabetic populations, HA-based treatments have been found to improve early soft-tissue healing, pain, and swelling parameters; however, the results remain heterogeneous across formulations [46,47,48,56,57]. Although available studies suggest that locally delivered HA-based interventions improve early post-extraction healing in type-2 diabetes, few have evaluated whether treatment response differs according to age and disease duration [35,36,50].

The present study shows that choice of treatment is significantly associated with improved early soft tissue healing trajectories in patients with T2DM post-tooth extraction. The results from the analysis were not confounded by age or years since diabetes diagnosis. Moreover, the improved healing trajectory observed with the six amino acid intervention suggests that differences in the amino acid composition may be clinically relevant. Although age and diabetes duration were included as covariates, residual confounding by other clinically relevant factors cannot be excluded. The association between glycemic control and dental-extraction outcomes remains incompletely defined when predicting impaired healing or increased risk of postoperative complications [28,59], while postoperative complications represent potential outcomes rather than baseline confounders. Both glycemic control and body mass indices should be evaluated prospectively in larger trials directly comparing AM4 and AM6. Furthermore, incorporating mechanistic endpoints into the study design may further shed light on the mechanism underlying treatment-healing response in diabetic extraction sockets [39].

## 5. Conclusions

The findings obtained in this pooled individual participant data analysis provide evidence that a novel hyaluronic acid-based adjunctive therapy supplemented with six selected amino acids significantly improves post-extraction socket healing in diabetes patients, after accounting for age and diabetes duration. Across interventions, modified healing indices improved in a predominantly linear fashion over time when compared with controls, and this pattern was independent of age and years since diabetes diagnosis. Importantly, the time × treatment trajectory showed a significantly greater improvement in the cohort treated with AM6 compared with AM4, indicating that this formulation promotes a faster recovery during socket repair after control-adjusted healing scores, with both interventions reaching comparable levels of healing repair by the end of the follow-up period. Overall, these results indicate that post-extraction healing in patients with diabetes is driven primarily by treatment choice, rather than by age or disease chronicity.

Clinically, our findings support the use of HA-based interventions to enhance post-extraction socket healing in patients with T2DM; supplementing this formulation with the six selected amino acids showed significantly faster socket healing recovery across time, with potential benefits in postoperative outcomes. The absence of a clear effect of age or years since diabetes diagnosis (diabetes duration) in healing suggests that the benefits of treating post-tooth extraction sockets with this HA-based adjunctive therapy may extend across a wide range of patients with T2DM in the dental surgery context. The main limitation of this study is the potential for residual confounding between the two pooled datasets, despite adjustment for participant-level covariates. Future research with two-arm randomized comparisons of both interventions is warranted to confirm the observed advantage of supplementing HA with the six selected amino acids to better inform the integration of this adjunctive strategy into post-tooth extraction care for individuals with diabetes.

## Figures and Tables

**Table 1:** Baseline demographic and clinical data across the four study groups. Data are expressed as mean ± SD (SD: Standard Deviation) or n (%).

	Values at Baseline	N
**Sex**		
Male	51%	57
Female	49%	55
**Ethnic origin**		
Caucasian	100%	112
**Age (years)**	69.44 ± 10.08	---
**Diabetes Duration** ^1^		
1–5 years	18%	21
6–10 years	35%	40
>10 years	47%	51
**Smokers**	33%	37
**BMI (kg/m^2^)** ^2^	28.2 ± 4.8	---
**Preoperative diabetes status**		
<180 mg/dL	82%	92
>180 mg/dL <240 mg/dL	16%	18
>240 mg/dL	2%	2
**HbA1c (%)** ^3^	7.3 ± 1.2 (mmol/mol)	---
**End-organ disease score**		
Cardiomyopathy	73%	82
Nephropathy	23%	26
Neuropathy	21%	23
Peripheral vasculopathy	28%	31
Retinopathy	10%	11
**Systemic risk**		
Low/Absent	9%	10
Moderate	56%	63
High	35%	39

^1^ Diabetes Duration: Years from first diabetes diagnosis to baseline assessment. ^2^ BMI: Body Mass Index. ^3^ HbA1c: Glycated hemoglobin.

**Table 2:** Modified healing index (mHI) descriptive statistics for the two intervention groups (AM6 and AM4) and their respective controls (C4 and C6) at Day 3, Day 7, Day 14, and Day 21. Values are presented as mean, standard deviation (SD), median, minimum, and maximum, with the number of participants (N) shown for each group and time point. AM4: group treated with Aminogam^®^ 4 gel; C4: control group corresponding to AM4; AM6: group treated with Aminogum^®^/Aminogam^®^ 6 Gel; C6: control group corresponding to AM6. * *p* < 0.05.

Modified Healing Index (mHI)					
Group		Day 3	Day 7	Day 14	Day 21
**AM4**	**N**	35	35	35	36
	**Mean**	6.49	5.00	4.09	4.00
	**SD**	1.634	1.213	0.507	0.000
	**Median**	6.00	5.00	4.00	4.00
	**Minimum**	4	4	4	4
	**Maximum**	10	9	7	4
**C4**	**N**	35	35	35	36
	**Mean**	7.63	5.91	4.54	4.00
	**SD**	1.750	1.669	1.010	0.000
	**Median**	8.00	5.00	4.00	4.00
	**Minimum**	5	4	4	4
	**Maximum**	11	9	7	4
**AM6**	**N**	21	21	21	21
	**Mean**	5.81	4.05 *	4.00 *	4.00
	**SD**	2.112	0.218	0.000	0.000
	**Median**	5.00	4.00	4.00	4.00
	**Minimum**	4	4	4	4
	**Maximum**	10	5	4	4
**C6**	**N**	19	19	19	19
	**Mean**	6.05	5.42	4.47	4.00
	**SD**	1.177	1.170	0.841	0.000
	**Median**	6.00	5.00	4.00	4.00
	**Minimum**	4	4	4	4
	**Maximum**	8	7	6	4

**Table 3:** Univariate analysis (ANOVA) comparing group AM6 with group AM4 at Day 3, Day 7, and Day 14. The table reports the between-group and within-group sums of squares, degrees of freedom (df), mean squares, F statistics, and associated *p*-values. AM4: group treated with Aminogam^®^ 4 gel; AM6: group treated with Aminogum^®^/Aminogam^®^ 6 Gel. ** *p* < 0.01.

Univariate Analysis (ANOVA)—(AM6 vs. AM4)						
AM6 vs. AM4		Sum of Squares	Df	Mean Square	F	*p*-Value
Day 3	Between Groups	6.001	1	6.001	1.801	0.185
	Within Groups	179.981	54	3.333	---	---
	Total	185.982	55	---	---	---
Day 7	Between Groups	11.905	1	11.905	12.617	0.001 **
	Within Groups	50.952	54	0.944	---	---
	Total	62.857	55	---	---	---
Day 14	Between Groups	0.096	1	0.096	0.596	0.444
	Within Groups	8.743	54	0.162	---	---
	Total	8.839	55	---	---	---

**Table 4:** Multivariate Analysis: Repeated-measures GLM assessing the effects of treatment over time on healing index scores, adjusting for age, across the four time points. **A**. Mauchly’s test of sphericity for the within-subjects factor time (four time points). Epsilon corrections (Greenhouse–Geisser, Huynh–Feldt, and lower-bound) are provided for violations of the sphericity assumption. **B**. Within-subjects effects from the repeated measures GLM on healing index scores. Sources include time, time × age, time × treatment, and error (time). Values shown are Type III sum of squares, degrees of freedom, mean square, F statistic, and significance (*p*-value). Sphericity-assumed values and corrections (Greenhouse–Geisser, Huynh–Feldt, and lower-bound) are reported. **C**. Planned contrasts of the within-subjects factor time from the repeated measures GLM. Sources include time, time × age, time × treatment, and error (time). Type III sum of squares, degrees of freedom, mean square, F statistic, and *p*-values are reported. **D**. Between-subjects effects (AM6 vs. AM4). AM4: group treated with Aminogam^®^ 4 gel; AM6: group treated with Aminogum^®^/Aminogam^®^ 6 Gel. * *p* < 0.05; ** *p* < 0.01.

**A. Mauchly’s Test of Sphericity**									
					**Epsilon **				
**Within Subjects**	**Mauchly’s W**	**Approx.** **Chi-Square**	**Df**	**Sign.**	**Greenhouse-** **Geisser**		**Huynh-** **Feldt**	**Lower-** **Bound**	
**Time**	0.098	120.062	5	0.000	0.495		0.525	0.333	
**B. Tests of Within-Subjects Effects**									
	**Source**		**Type III Sum** **of Squares**		**Df**	**Mean** **Square**		**F**	**Sig.**
Time	Sphericity assumed		7.595		3	2.532		2.630	0.052
	Greenhouse–Geisser		7.595		1.484	5.118		2.630	0.093
	Huynh–Feldt		7.595		1.574	4.825		2.630	0.090
	Lower-bound		7.595		1.000	7.595		2.630	0.111
Time × Age	Sphericity assumed		9.846		3	3.282		3.410	0.019
	Greenhouse–Geisser		9.846		1.484	6.636		3.410	0.052
	Huynh–Feldt		9.846		1.574	6.255		3.410	0.049
	Lower-bound		9.846		1.000	9.846		3.410	0.070
Time × Treatment	Sphericity assumed		16.528		3	5.509		5.724	0.001
	Greenhouse–Geisser		16.528		1.484	11.139		5.724	0.010 *
	Huynh–Feldt		16.528		1.574	10.500		5.724	0.008 **
	Lower-bound		16.528		1.000	16.528		5.724	0.020 *
Error (time)	Sphericity assumed		153.044		159	0.963		---	---
	Greenhouse–Geisser		153.044		78.643	1.946		---	---
	Huynh–Feldt		153.044		83.431	1.834		---	---
	Lower-bound		153.044		53.000	2.888		---	---
**C. Tests of Within-Subjects Contrasts**									
**Source**		**mHI**	**Type III Sum of Squares**		**Df**	**Mean** **Square**		**F**	**Sig.**
Time		Linear	0.453		1	0.453		0.276	0.602
Time × age		Linear	4.399		1	4.399		2.681	0.107
Time × treatment		Linear	9.564		1	9.564		5.828	0.019 *
Error (Time)		Linear	86.971		53	1.641		---	---
**D. Tests of Between-Subjects Effects**									
**Source**		**Type III Sum of Squares**	**Df**		**Mean** **Square**	**F**		**Sig.**	
Intercept		40.034	1		40.034	29.493		0.000	
Age		4.843	1		4.843	3.568		0.064	
Treatment		14.411	1		14.411	10.616		0.002 **	
Error		71.942	53		1.357	---		---	

**Table 5:** Multivariate Analysis: Repeated-measures GLM assessing the effects of treatment (AM6 vs. AM4) on healing index scores relative to their respective controls (C6, C4), adjusting for age, across the four time points. **A**. Mauchly’s test of sphericity for the within-subjects factor time (four time points). Epsilon corrections (Greenhouse–Geisser, Huynh–Feldt, and lower-bound) are provided for violations of the sphericity assumption. **B**. Within-subjects effects from the repeated measures GLM on healing index scores. Sources include time, time × age, time × treatment, and error (time). Values shown are the Type III sum of squares, degrees of freedom, mean square, F statistic, and significance (*p*-value). Sphericity-assumed values and corrections (Greenhouse–Geisser, Huynh–Feldt, and lower-bound) are reported. **C**. Planned contrasts of the within-subjects factor time from the repeated measures GLM. Sources include time, time × age, time × treatment, and error (time). Type III sum of squares, degrees of freedom, mean square, F statistic, and *p*-values are reported. **D**. Between-Subjects Effects (AM6 vs. AM4). AM4: group treated with Aminogam^®^ 4 gel; AM6: group treated with Aminogum^®^/Aminogam^®^ 6 Gel. * *p* < 0.05; ** *p* < 0.01; *** *p* < 0.001.

**A. Mauchly’s Test of Sphericity**									
					**Epsilon**				
**Within Subjects**	**Mauchly’s W**	**Approx.** **Chi-Square**	**Df**	**Sign.**	**Greenhouse-** **Geisser**		**Huynh-** **Feldt**	**Lower-** **Bound**	
Time	0.364	104.854	5	0.000	0.656		0.694	0.333	
**B. Tests of Within-Subjects Effects**									
	**Source**		**Type III Sum** **of Squares**		**Df**	**Mean** **Square**		**F**	**Sig.**
Time	Sphericity assumed		2.842		3	0.947		0.912	0.435
	Greenhouse–Geisser		2.842		1.968	1.444		0.912	0.402
	Huynh–Feldt		2.842		2.082	1.365		0.912	0.406
	Lower-bound		2.842		1.000	2.842		0.912	0.342
Time × Age	Sphericity assumed		7.898		3	2.633		2.536	0.057
	Greenhouse–Geisser		7.898		1.968	4.013		2.536	0.083
	Huynh–Feldt		7.898		2.082	3.794		2.536	0.079
	Lower-bound		7.898		1.000	7.898		2.536	0.114
Time × Treatment	Sphericity assumed		54.378		9	6.042		5.821	0.000
	Greenhouse–Geisser		54.378		5.904	9.210		5.821	0.000 ***
	Huynh–Feldt		54.378		6.245	8.707		5.821	0.000 ***
	Lower-bound		54.378		3.000	18.126		5.821	0.001 **
Error (time)	Sphericity Assumed		326.986		315	1.038		---	---
	Greenhouse-Geisser		326.986		206.647	1.582		---	---
	Huynh-Feldt		326.986		218.585	1.496		---	---
	Lower-bound		326.986		105.000	3.114		---	---
**C. Tests of Within-Subjects Contrasts**									
**Source**		**mHI**	**Type III Sum of Squares**		**Df**	**Mean** **Square**		**F**	**Sig.**
Time		Linear	8.593 × 10^−7^		1	8.593 × 10^−7^		0.000	0.999
Time × age		Linear	6.390		1	6.390		4.304	0.040 *
Time × treatment		Linear	41.750		3	13.917		9.373	0.000 ***
Error (Time)		Linear	155.904		105	1.485		---	---
**D. Tests of Between-Subjects Effects**									
**Source**		**Type III Sum of Squares**	**Df**		**Mean** **Square**	**F**		**Sig.**	
Intercept		100.546	1		100.546	55.819		0.000 ***	
Age		11.572	1		11.572	6.424		0.013 *	
Treatment		74.722	3		24.907	13.827		0.000 ***	
Error		189.137	105		1.801	---		---	

**Table 6:** Multivariate Analysis: Repeated-measures GLM assessing the effects of treatment (AM6 vs. AM4) on healing index scores adjusting for years since T2DM diagnosis (T2DM Dx), across the four time points. **A**. Mauchly’s test of sphericity for the within-subjects factor time (4 time points). Epsilon corrections (Greenhouse–Geisser, Huynh–Feldt, and lower-bound) are provided for violations of the sphericity assumption. **B**. Within-subjects effects from the repeated measures GLM on healing index scores. Sources include time, time × T2DM Dx, time × treatment, and error (time). Values shown are Type III sum of squares, degrees of freedom, mean square, F statistic, and significance (*p*-value). Sphericity-assumed values and corrections (Greenhouse–Geisser, Huynh–Feldt, and lower-bound) are reported. **C**. Planned contrasts of the within-subjects factor time from the repeated-measures GLM. Sources include time, time × T2DM Dx, time × treatment, and error (Time). Type III sum of squares, degrees of freedom, mean square, F statistic, and *p*-values are reported. **D**. Between-subjects effects (AM6 vs. AM4). AM4: group treated with Aminogam^®^ 4 gel; AM6: group treated with Aminogum^®^/Aminogam^®^ 6 Gel. * *p* < 0.05; ** *p* < 0.01; *** *p* < 0.001.

**A. Mauchly’s Test of Sphericity**									
					**Epsilon**				
**Within Subjects**	**Mauchly’s W**	**Approx.** **Chi-Square**	**Df**	**Sign.**	**Greenhouse-** **Geisser**		**Huynh-** **Feldt**	**Lower-** **Bound**	
**Time**	0.112	111.030	5	0.000	0.535		0.571	0.333	
**B. Tests of Within-Subjects Effects**									
	**Source**		**Type III Sum** **of Squares**		**Df**	**Mean** **Square**		**F**	**Sig.**
Time	Sphericity assumed		30.098		3	10.033		10.000	---
	Greenhouse–Geisser		30.098		1.605	18.756		10.000	0.000 ***
	Huynh–Feldt		30.098		1.712	17.584		10.000	0.000 ***
	Lower-bound		30.098		1.000	30.098		10.000	0.003 **
Time × T2DM Dx	Sphericity assumed		1.391		3	0.464		0.462	0.709
	Greenhouse–Geisser		1.391		1.605	0.867		0.462	0.589
	Huynh–Feldt		1.391		1.712	0.812		0.462	0.601
	Lower-bound		1.391		1.000	1.391		0.462	0.500
Time × Treatment	Sphericity assumed		8.399		3	2.800		2.791	0.042
	Greenhouse–Geisser		8.399		1.605	5.234		2.791	0.078
	Huynh–Feldt		8.399		1.712	4.907		2.791	0.075
	Lower-bound		8.399		1.000	8.399		2.791	0.101
**Error (time)**	Sphericity assumed		156.503		156	1.003		---	---
	Greenhouse–Geisser		156.503		83.445	1.876		---	---
	Huynh–Feldt		156.503		89.006	1.758		---	---
	Lower-bound		156.503		52.000	3.010		---	---
**C. Tests of Within-Subjects Contrasts**									
**Source**		**mHI**	**Type III Sum of Squares**		**Df**	**Mean** **Square**		**F**	**Sig.**
Time		Linear	21.027		1	21.027		12.373	0.001 **
Time × T2DM Dx		Linear	0.006		1	0.006		0.003	0.954
Time × treatment		Linear	3.968		1	3.968		2.335	0.133
Error (time)		Linear	88.370		52	1.699		---	---
**D. Tests of Between-Subjects Effects**									
**Source**		**Type III Sum of Squares**	**Df**		**Mean** **Square**	**F**		**Sig.**	
Intercept		820.561	1		820.561	567.013		0.000	
T2DM Dx		0.015	1		0.015	0.011		0.918	
Treatment		7.004	1		7.004	4.840		0.032 *	
Error		75.253	52		1.447	---		---	

**Table 7:** Multivariate Analysis: Repeated-measures GLM assessing the effects of treatment (AM6 = AM4) on healing index scores relative to their respective controls, adjusting for years since diabetes diagnosis (T2DM Dx), across the four time points. **A**. Mauchly’s test of sphericity for the within-subjects factor time (four time points). Epsilon corrections (Greenhouse–Geisser, Huynh–Feldt, and lower-bound) are provided for violations of the sphericity assumption. **B**. Within-subjects effects from the repeated measures GLM on healing index scores. Sources include time, time × T2DM Dx, time × treatment, and error (time). Values shown are Type III sum of squares, degrees of freedom, mean square, F statistic, and significance (*p*-value). Sphericity-assumed values and corrections (Greenhouse–Geisser, Huynh–Feldt, and lower-bound) are reported. **C**. Planned contrasts of the within-subjects factor time from the repeated measures GLM. Sources include time, time × T2DM Dx, time × treatment, and error (time). Type III sum of squares, degrees of freedom, mean square, F statistic, and *p*-values are reported. **D**. Between-subjects effects (AM6 vs. AM4). AM4: group treated with Aminogam^®^ 4 gel; AM6: group treated with Aminogum^®^/Aminogam^®^ 6 Gel. ** *p* < 0.01; *** *p* < 0.001.

**A. Mauchly’s Test of Sphericity**									
					**Epsilon**				
**Within Subjects**	**Mauchly’s W**	**Approx.** **Chi-Square**	**Df**	**Sign.**	**Greenhouse–** **Geisser**		**Huynh–** **Feldt**	**Lower-** **Bound**	
**Time**	0.375	99.700	5	0.000	0.670		0.710	0.333	
**B. Tests of Within-Subjects Effects**									
	**Source**		**Type III Sum** **of Squares**		**Df**	**Mean** **Square**		**F**	**Sig.**
**Time**	Sphericity assumed		80.209		3	26.736		25.425	0.000
	Greenhouse–Geisser		80.209		2.009	39.921		25.425	0.000 ***
	Huynh–Feldt		80.209		2.129	37.678		25.425	0.000 ***
	Lower-bound		80.209		1.000	80.209		25.425	0.000 ***
**Time ×** **T2DM Dx**	Sphericity assumed		0.412		3	0.137		0.131	0.942
	Greenhouse–Geisser		0.412		2.009	0.205		0.131	0.878
	Huynh–Feldt		0.412		2.129	0.194		0.131	0.889
	Lower-bound		0.412		1.000	0.412		0.131	0.718
**Time ×** **Treatment**	Sphericity assumed		46.747		9	5.194		4.939	0.000
	Greenhouse–Geisser		46.747		6.028	7.756		4.939	0.000 ***
	Huynh–Feldt		46.747		6.386	7.320		4.939	0.000 ***
	Lower-bound		46.747		3.000	15.582		4.939	0.003 **
**Error (time)**	Sphericity assumed		324.942		309	1.052		---	---
	Greenhouse–Geisser		324.942		206.946	1.570		---	---
	Huynh–Feldt		324.942		219.269	1.482		---	---
	Lower-bound		324.942		103.000	3.155		---	---
**C. Tests of Within-Subjects Contrasts**									
**Source**		**mHI**	**Type III Sum of Squares**		**Df**	**Mean** **Square**		**F**	**Sig.**
**Time**		Linear	72.076		1	72.076		47.938	0.000 ***
**Time × T2DM Dx**		Linear	0.026		1	0.026		0.017	0.896
**Time × treatment**		Linear	35.435		3	11.812		7.856	0.000 ***
**Error (Time)**		Linear	154.862		103	1.504		---	---
**D. Tests of Between-Subjects Effects**									
**Source**		**Type III Sum of Squares**	**Df**		**Mean** **Square**	**F**		**Sig.**	
**Intercept**		1996.770	1		1996.770	1068.233		0.000 ***	
**T2DM Dx**		0.004	1		0.004	0.002		0.964	
**Treatment**		63.946	3		21.315	11.403		0.000 ***	
**Error**		192.530	103		1.869	-		-	

## Data Availability

The original contributions presented in this study are included in the article. Further inquiries can be directed to the corresponding authors.

## Supplementary Materials

The following supporting information can be downloaded at: https://www.mdpi.com/article/10.3390/jfb17080354/s1, Table S1: Participant age (years) presented as means by group; Table S2: Genre distribution by group presented as % (*n*); Table S3: Body mass index (BMI) expressed in kg/m^2^, presented as means by group; Table S4: Years since diagnosis by group (1–5, 6–10, >10 years), presented as % (n).

## Abbreviations

The following abbreviations are used in this manuscript:

T2DM	Type 2 diabetes mellitus
HA	Hyaluronate
BMI	Body Mass Index
HbA1c	Glycated Hemoglobin
