# Supplementary material for: Healing Response to Hyaluronic Acid Supplemented with Selected Amino Acids: A Pooled Analysis of Two Clinical Studies
Source: J Funct Biomater. 2026 Jul 24;17(8):354. doi: 10.3390/jfb17080354 (PMC13514193; doi:10.3390/jfb17080354)
Supplement: Supplementary file 1 [file jfb-17-00354-s001.zip › jfb-4393680-supplementary.pdf]

Healing response to Hyaluronic Acid supplemented with selected Amino Acids: A pooled analysis of two clinical studies

Tiziana Ruggiero, Marta Bezzi, Ettore Cogno, Davide Camisassa, Renato Pol, Francesco Erovigni, Giorgio Reggiardo, Vincenzo Nobile and Paolo Giacomo Arduino

Table S1. Participant age (years) presented as means by group: descriptive statistics and between-group ANOVA. AM4: group treated with Aminogam® 4 gel; C4: group control of AM4; AM6: group treated with Aminogum®/Aminogam® 6 Gel; C6: group control of AM6.

Age (years) at baseline						
**Group**	**N**	**Mean**	**S.D.** [1]	**Median**	**Minimum**	**Maximum**
AM4	36	66.853	10.9739	67.585	42.8	86.1
C4	36	66.853	10.9739	67.585	42.8	86.1
AM6	21	78.088	3.3700	78.841	71.8	81.3
C6	19	69.686	5.6491	74.416	61.9	75.7
TOTAL	112	69.440	10.0831	71.844	42.8	86.1
ANOVA Between Groups *p-value* < **0.001**						

[1] S.D.: Standard Deviation

Table S2. Genre distribution by group presented as % (n). Between-group differences assessed with chi-square test. AM4: group treated with Aminogam® 4 gel; C4: group control of AM4; AM6: group treated with Aminogum®/Aminogam® 6 Gel; C6: group control of AM6.

Genre distribution (%) at baseline			
**Group**	**Female % (n)**	**Male % (n)**	**Total % (n)**
AM4	52.8% (19)	47.2% (17)	100.0% (36)
C4	52.8% (19)	47.2% (17)	100.0% (36)
AM6	71.4% (15)	28.6% (6)	100.0% (21)
C6	10.5% (2)	89.5% (17)	100.0% (19)
TOTAL	49.1% (55)	50.9% (57)	100.0% (112)
Chi Square test *p-value* = **0.001**			

Healing response to Hyaluronic Acid supplemented with selected Amino Acids: A pooled analysis of two clinical studies

Tiziana Ruggiero, Marta Bezzi, Ettore Cogno, Davide Camisassa, Renato Pol, Francesco Erovigni, Giorgio Reggiardo, Vincenzo Nobile and Paolo Giacomo Arduino

Table S3. Body mass index (BMI) expressed in kg/m², presented as means by group: descriptive statistics and between-group ANOVA. AM4: group treated with Aminogam® 4 gel; C4: group control of AM4; AM6: group treated with Aminogum®/Aminogam® 6 Gel; C6: group control of AM6.

**Body Mass Index (BMI, kg/m²) at baseline**						
**Group**	**N**	**Mean**	**S.D.** [1]	**Median**	**Minimum**	**Maximum**
**AM4**	33	28.2109	4.82285	28.6000	22.00	39.90
**C4**	33	28.2109	4.82285	28.6000	22.00	39.90
**AM6**	21	28.2386	5.83549	33.1500	19.92	34.19
**C6**	19	33.1137	9.12739	27.0400	24.20	46.98
**TOTAL**	106	29.0952	6.20529	28.6000	19.92	46.98
ANOVA Between Groups *p-value* = 0.019						

[1] S.D.: Standard Deviation

Table S4. Years since T2DM diagnosis by group (1–5, 6–10, >10 years), presented as %(n); between-group differences assessed with chi-square test. AM4: group treated with Aminogam® 4 gel; C4: group control of AM4; AM6: group treated with Aminogum®/Aminogam® 6 Gel; C6: group control of AM6.

**% (n) grouped by T2DM Dx (Ýears since T2DM diagnosis at baseline)**				
**Group**	**1–5 y %(n)**	**6–10 y %(n)**	**> 10 y %(n)**	**Total %(n)**
**AM4**	25.7% (9)	37.1% (13)	37.1% (13)	100.0% (35)
**C4**	25.7% (9)	37.1% (13)	37.1% (13)	100.0% (35)
**AM6**	0.0% (0)	23.8% (5)	76.2% (16)	100.0% (21)
**C6**	0.0% (0)	47.4% (9)	52.6% (10)	100.0% (19)
**TOTAL**	16.4% (18)	36.4% (40)	47.3% (52)	100.0% (110)
Chi Square test *p-value* = **0.009**				
